# Transactions of the Obstetrical Society of London, for the Year 1860

**Published:** 1861-11

**Authors:** 


ART. XI.—*Transactions of the Obstetrical Society of London,* vol. ii. for the year 1860. 8vo., pp. 368. London, 1861.

THE volume before us contains many interesting papers upon facts and theories of obstetrical science and art. We have here recorded cases of labor impeded by unnatural growths, ovarian tumors, lacerations of the cervix uteri, monstrosities, ruptured perinæums, and many of the accidents and annoyances which give pain to the sufferer, and anxiety to the practitioner of midwifery. Several of these are especially interesting, but we have space for mere allusion to them, and cannot indulge in a notice, which we should otherwise have given, of a paper in these Transactions, by Dr. A. B. Granville, on "certain phenomena, facts, and calculations incidental to or connected with the power and act of propagation in females of the industrial classes in the metropolis." The other papers seem well chosen, and the whole collection forms a very readable and instructive book, in which those interested in obstetrics may peruse much that is novel and curious.

